# Imaging Salt Uptake Dynamics in Plants Using PET

**DOI:** 10.1038/s41598-019-54781-z

**Published:** 2019-12-09

**Authors:** Gerard Ariño-Estrada, Gregory S. Mitchell, Prasenjit Saha, Ahmad Arzani, Simon R. Cherry, Eduardo Blumwald, Andre Z. Kyme

**Affiliations:** 1 Department of Biomedical Engineering, University of California Davis, Davis, CA USA; 2 Calyxt, Inc., Roseville, MN USA; 3 Deparment of Plant Sciences, University of California Davis, Davis, CA USA; 4 Department of Agronomy and Plant Breeding, College of Agriculture, Isfahan University of Technology, Isfahan, Iran; 5 School of Biomedical Engineering, Faculty of Engineering & IT, University of Sydney, Sydney, Australia

**Keywords:** Natural variation in plants, Applied physics

## Abstract

Soil salinity is a global environmental challenge for crop production. Understanding the uptake and transport properties of salt in plants is crucial to evaluate their potential for growth in high salinity soils and as a basis for engineering varieties with increased salt tolerance. Positron emission tomography (PET), traditionally used in medical and animal imaging applications for assessing and quantifying the dynamic bio-distribution of molecular species, has the potential to provide useful measurements of salt transport dynamics in an intact plant. Here we report on the feasibility of studying the dynamic transport of ^22^Na in millet using PET. Twenty-four green foxtail *(Setaria viridis L. Beauv.)* plants, 12 of each of two different accessions, were incubated in a growth solution containing ^22^Na^+^ ions and imaged at 5 time points over a 2-week period using a high-resolution small animal PET scanner. The reconstructed PET images showed clear evidence of sodium transport throughout the whole plant over time. Quantitative region-of-interest analysis of the PET data confirmed a strong correlation between total ^22^Na activity in the plants and time. Our results showed consistent salt transport dynamics within plants of the same variety and important differences between the accessions. These differences were corroborated by independent measurement of Na^+^ content and expression of the *NHX* transcript, a gene implicated in sodium transport. Our results demonstrate that PET can be used to quantitatively evaluate the transport of sodium in plants over time and, potentially, to discern differing salt-tolerance properties between plant varieties. In this paper, we also address the practical radiation safety aspects of working with ^22^Na in the context of plant imaging and describe a robust pipeline for handling and incubating plants. We conclude that PET is a promising and practical candidate technology to complement more traditional salt analysis methods and provide insights into systems-level salt transport mechanisms in intact plants.

## Introduction

Soil salinity is a global environmental challenge for crop production, affecting over 800 million hectares or nearly a third of the earth’s agricultural land^1,2^. The dominant salt in saline soils is sodium chloride (NaCl), and the sodium component of NaCl is principally responsible for saline toxicity in plants. Saline toxicity is thought to be caused by the osmotic and ionic stresses exerted by elevated Na^+^ ion concentration in addition to downstream oxidative stresses that perturb cellular function and signaling^3,4^.

Given the scale of the soil salinity problem, increased salt tolerance is a highly desirable trait and a key attribute to be studied among crop plants adapted to marginal environments. Accordingly, several decades of research have focused on understanding and characterizing the sodium transport and distribution mechanisms underlying sodium tolerance^5–11^. However, current techniques tend to focus on the cellular basis of transport and there is much still to learn about sodium fluxes and regulation at the level of the whole plant. To better understand how sodium is distributed and regulated within whole organs and the whole plant, the development of novel methods to visualize and quantify sodium transport throughout entire intact plants would be a significant advance.

Several studies have tried to evaluate sodium transport using radioactive ^22^Na uptake from the nutrient solution^9,12–15^. However, the quantification approach used was destructive and unable to show the transport behavior throughout the intact plant. These studies also present contradictory reports regarding, for example, the correlation between salt tolerance and Na^+^ transport.

Plant traits related to growth and adaptation to biotic or abiotic stress have been studied using diverse imaging approaches, including fluorescence imaging and magnetic resonance imaging^16,17^. *In vivo* molecular imaging techniques such as gamma camera imaging and positron emission tomography (PET) have been extremely successful in studying biological processes non-invasively in animals and humans^18,19^ but have had minimal use in studying the transport of particular ions or compounds in plants. PET enables the uptake, utilization and excretion of radiolabeled molecules to be studied in the same individual or specimen over time. Combined with kinetic modelling methods, PET also allows derivation of important functional parameters characterizing molecular interactions (e.g. receptor-ligand binding). For these reasons there is growing interest in using PET and gamma cameras to image plants with appropriate radiotracers^20–22^.

PET relies on the detection of paired 511 keV gamma photons emitted from β^+^-emitting nuclei such as ^11^C, ^18^F, ^13^N, ^15^O and ^22^Na. A raw PET dataset typically comprises many millions of such events, each defining a line in space between detector elements of the PET system. By mathematically reconstructing these lines, an estimate of the 3D bio-distribution of the radiotracer can be computed. Importantly, with appropriate calibrations and corrections (e.g. for photon attenuation in the object), reconstructed PET images are quantitative. Moreover, a PET acquisition can be framed into a sequence of time segments allowing the dynamic behavior of the tracer to be visualized and quantified^23^.

Due to the global interest in plant productivity in elevated levels of atmospheric carbon, carbon sequestration in biomass, and biofuel development, recent plant PET studies have mostly involved the short-lived ^11^C-labeled CO_2_ radiotracer to characterize the extraction and transport of carbon from the air into plants during photosynthesis^20,21,24^. However, it has also been recognized that xylem water flow can be imaged using ^15^O or ^18^F, and phloem sugar flow using ^11^C or ^18^F^25^. To our knowledge there is only one published study using PET to image sodium transport characteristics in intact plants with ^22^Na-labeled NaCl^26^. There the authors investigated the steady-state bio-distribution of ^22^Na in common reed and rice using a parallel-plate PET system and found significant differences in Na^+^ concentrations in roots compared to upper shoots, suggesting the existence of an exclusion mechanism in common reed that was not present in rice. However, this study used a limited angle tomography system and only reported dynamic salt transport for 18 h post feeding.

In this paper, we build on the promising work in^17^ and^26^ by considering the dynamic transport of ^22^Na in plants over a 2-week period using a high resolution full-ring PET system for 3D imaging. We hypothesize that dynamic PET imaging of sodium over physiologically relevant timescales using the long half-life ^22^Na radioisotope has the potential to reveal important aspects of macroscopic sodium transport in intact plants which corroborate and build on observations obtained using other methods. Moreover, preclinical PET scanners could potentially provide an efficient screening tool for identifying salt-tolerant genotypes. We evaluate our hypothesis here using ^22^Na PET imaging in two contrasting varieties of green foxtail millet. Green foxtail is found in a variety of harsh environments worldwide. Its wide geographical distribution and enormous genetic diversity explain its adaptability to many environmental conditions and make it an ideal germplasm for abiotic stress studies^11,27^. Green foxtail is also of interest because it uses C_4_ carbon fixation during photosynthesis. Currently plants with C_4_ photosynthesis predominately occupy arid regions with dry and hot environmental conditions^28^, however global climate change could result in the expansion of C_4_ plants into rangeland and grassland ecosystems. Our PET analysis of the two green foxtail varieties was cross-validated against conventional measurements of Na^+^ content and the expression of *NHX* transcript, a gene implicated in sodium transport^29^.

## Materials and Methods

### Plant material

We used two accessions of green-foxtail (*Setaria viridis*), Ast-1 and A10.1. In previous work, the Ast-1 genotype (PI 223677) was shown to be highly sensitive to water deficit and heat stresses compared to the A10.1 genotype (PI 669942)^27^. Seeds of both accessions were de-husked and surface sterilized according to^30^ and grown in half strength Murashige and Skoog (MS) growth medium^31^. Seedlings were grown individually in a growth chamber for 14 days in plastic cups prior to entering experiments.

### Plant preparation

Twelve plants of each accession were incubated for 330 h (approximately 14 days) in a solution containing 10 mL half strength MS growth medium supplemented with 185 kBq (5 µCi) of ^22^Na in 50 μL. The total concentration of Na^+^ (cold and hot) in the incubation solution was 1.65 mM, well below the concentration expected to cause salt stress in these seedlings. Therefore, in this study we were observing salt transport under physiologically normal (non-stress and non-toxic) salt conditions. Plants were maintained at room temperature on a 16 h:8 h light:dark cycle.

**Figure 1:**
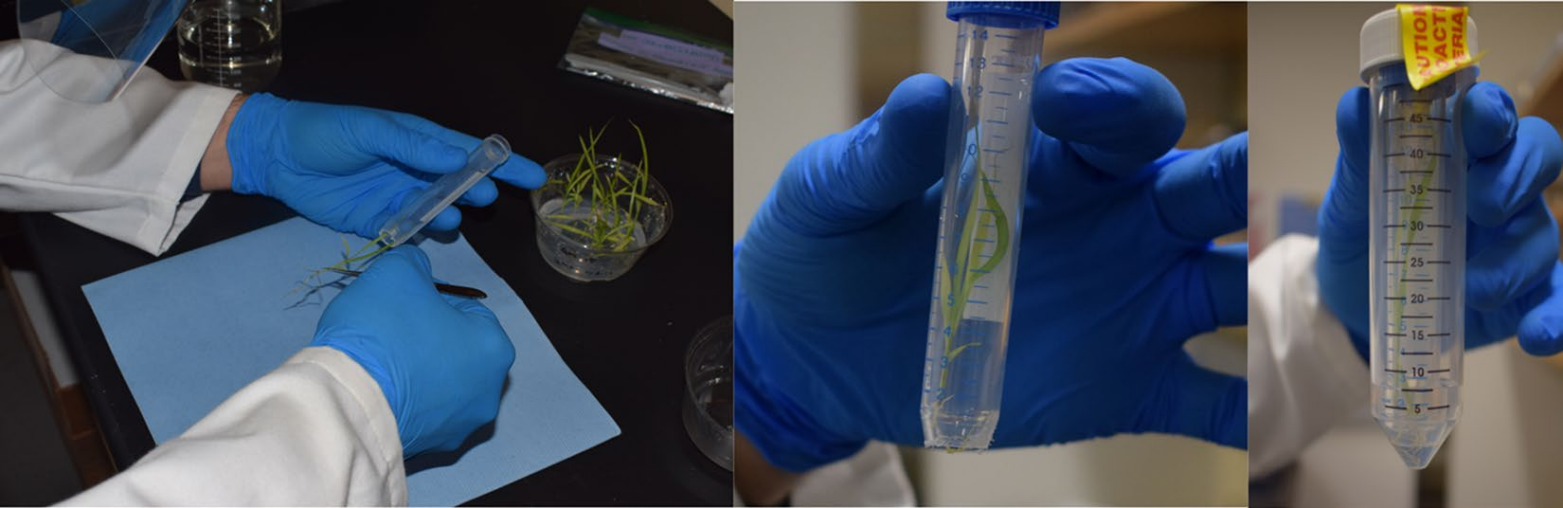
Each plant was kept inside a 15 mL tube with the base removed (left and center). This tube was placed inside a larger (50 mL) tube containing the incubation solution (right).

Since radiation safety is a key concern when working with ^22^Na due to its long physical half-life (2.6 years), each plant was kept inside a 15 mL tube isolated inside a larger 50 mL tube, as shown in Fig. 1. The plants were never removed from the 15 mL tube to minimize potential contamination of the shoots and laboratory environment during manipulation.

Approximately 10 mm of the base of the 15 mL tube was removed (Fig. 1, left and center) to allow the roots to soak in the incubation solution in the base of the larger tube (Fig. 1, right). Immediately prior to each imaging time point, the 15 mL tube containing the plant was removed from the incubation solution so that the roots could be rinsed with water to remove residual ^22^Na. The radioactivity in the plant, growth media and rinsing water was measured using a well counter prior to each imaging session to track the residual activity, potential spills and contamination. Plants were also photographed in a reproducible position before each imaging session to enable co-registration with the reconstructed PET images during analysis.

### Imaging

**Figure 2:**
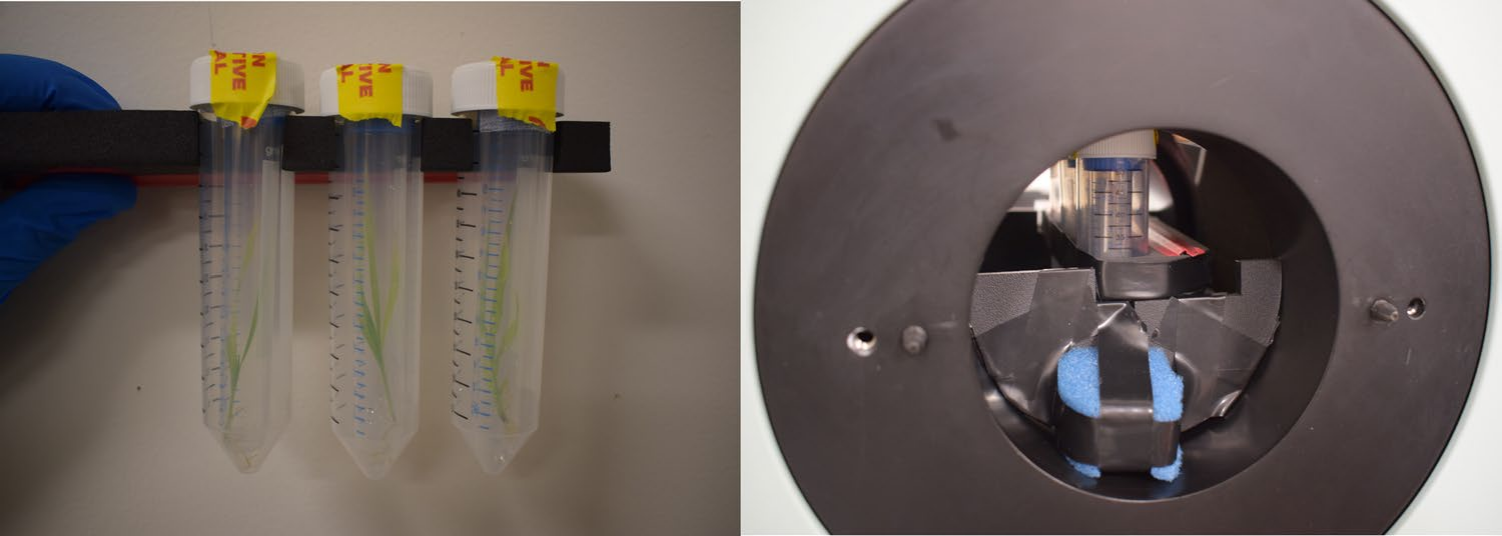
(Left) Set of three plants reproducibly positioned in a foam holder for imaging. (Right) Set of three plants being imaged inside the small animal PET scanner.

For imaging, each plant (inside the 15 mL tube) was placed inside a clean and dry 50 mL tube to provide secondary containment. Imaging was performed on the Inveon small animal PET scanner (Preclinical Solutions, Siemens Healthcare Molecular Imaging). The Inveon has a bore diameter of 12 cm and a trans-axial and axial field-of-view (FoV) of 10 cm and 12.7 cm, respectively. The radial, tangential and axial spatial resolution at the center of the FoV is 1.46 mm, 1.49 mm and 1.15 mm, respectively, and the coincidence photon detection sensitivity is 5.75% for an energy window of 350–650 keV^32^. Plants were imaged three at a time for 20 min per scan in a reproducible position and orientation (Fig. 2). Therefore, 8 scans were required per time point to image all 24 plants. Scans were repeated at *t* = 16 h, 64 h, 114 h, 234 h and 330 h (i.e. approximately 1, 3, 5, 10 and 14 days after the start of incubation). Plants were returned to the incubation media between imaging sessions.

### PET data analysis

PET images were reconstructed for each plant at each time point using a vendor-supplied OSEM3D/MAP algorithm (matrix size 128 × 128 × 159 pixels, reconstructed trans-axial pixel size 0.776 mm). The reconstructed PET images were first assessed qualitatively for evidence of sodium uptake and transport over the 2-week period. Images were overlaid with co-registered photographs to compare the physical plant structures with the measured activity distribution.

**Figure 3:**
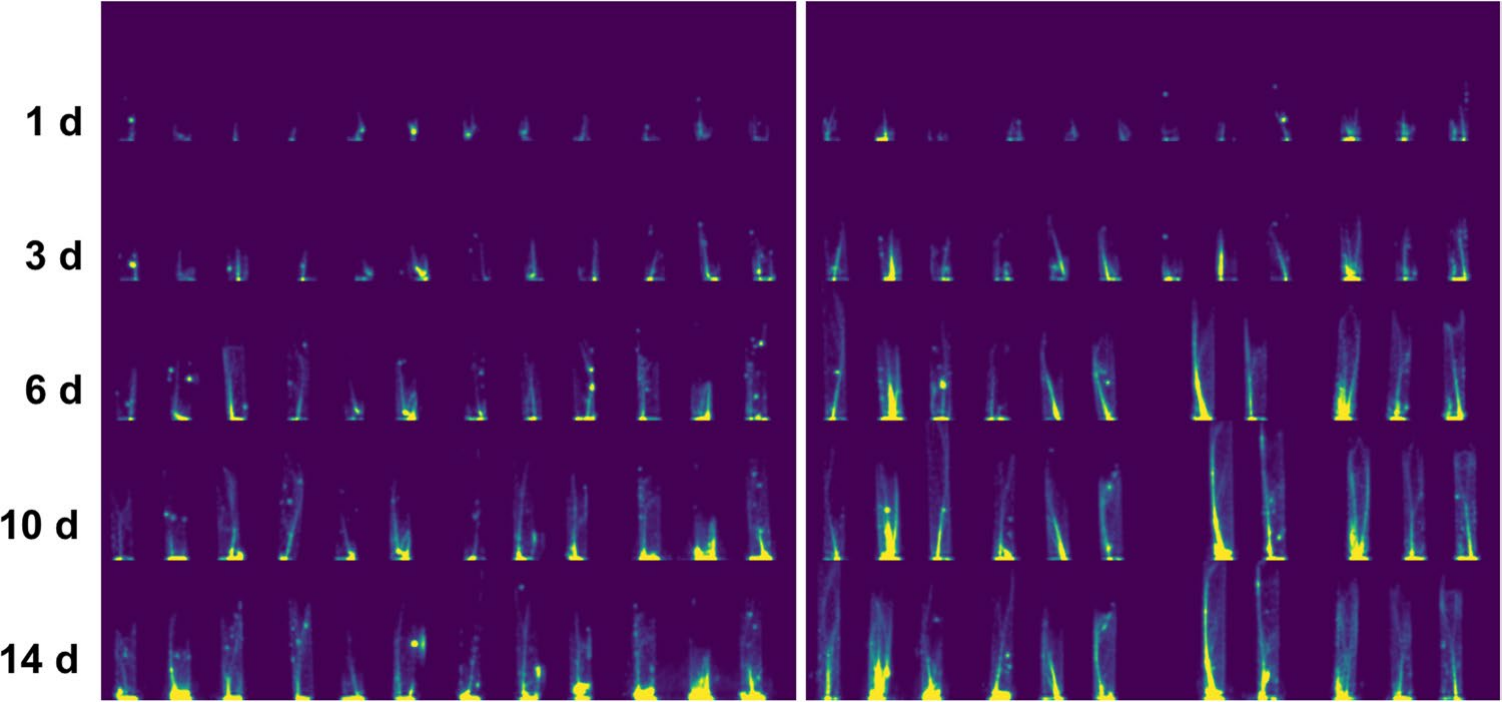
Maximum intensity projection (MIP) PET images showing ^22^Na uptake in the Ast-1 (left panel) and A10.1 (right panel) accessions. Rows (top to bottom) correspond to the time after incubation, in days. Columns (left to right) correspond to plants 1 to 24 (12 in each accession). Note that plant 21 (from the A10.1 dataset) was removed from the study after the second time point and is therefore missing from rows 3, 4 and 5 in the right panel.

Quantitative evaluation of sodium uptake was performed using a region-of-interest (ROI) analysis. Regions were drawn slice-by-slice around the cross-section of the 15 mL tube in the coronal (cross-sectional) plane. These regions excluded occasional instances of ^22^Na contamination on the sides of the tube, which were easily identified in the PET images as focal spots of activity coinciding with the tube wall (see Fig. 3). We also performed two-way analysis of variance (ANOVA) on the ROI data to analyze correlations between ^22^Na activity, time and plant variety.

### Dry weight measurements and gene expression analysis

To independently validate differences observed between the *Setaria viridis* accessions using PET, we compared the sodium content and the expression of *NHX* transcript, a gene implicated in sodium transport^29^, in seedlings using conventional methods. Approximately 50 seeds of both accessions, A10.1 and Ast-1, were first de-husked and surface sterilized according to^30^. The seedlings were then grown for 14 days in plastic cups before being transferred to 15 mL falcon tubes containing liquid half MS growth medium. Twenty-four days after germination (i.e. on day 10 according to the PET imaging timeline), the root and shoot of each seedling were separated. For Na^+^ content, roots and shoots were oven dried for 3 days at 70 °C and then the tissue was scaled, grinded, digested with nitric acid and hydrogen peroxide and finally analyzed using Inductively Coupled Plasma Atomic Emission Spectrometry (ICPAES)^33^. The amount of Na^+^ was determined as a percentage of dry weight. For gene expression analysis, shoot samples were first processed according to^27^, then qRT-PCR was performed according to^34^ to determine the relative expression of *NHX* transcript.

## Results

### PET imaging

Maximum intensity projection (MIP) PET images are shown in Fig. 3 for all plants and for every time point. There is clear evidence of ^22^Na uptake and progression from roots to shoots over time for both accessions of green foxtail. Activity is expected to be highest at the base of the images in Fig. 3 since this is the point of entry of sodium into the roots from the incubation solution. Higher activity in this part of the image may also be due in part to residual radioactivity on the surface of the roots post rinsing.

Some images in Fig. 3 (e.g. column 6, row 5) exhibited isolated focal spots of activity, likely due to splashes of ^22^Na on either the inner or outer tube during rinsing, or the removal/replacement of plants in the incubation solution before/after an imaging session. These instances of contamination were excluded from the quantification analysis. Plant 21 was removed from the experiment after 6 days because of a handling problem.

**Figure 4:**
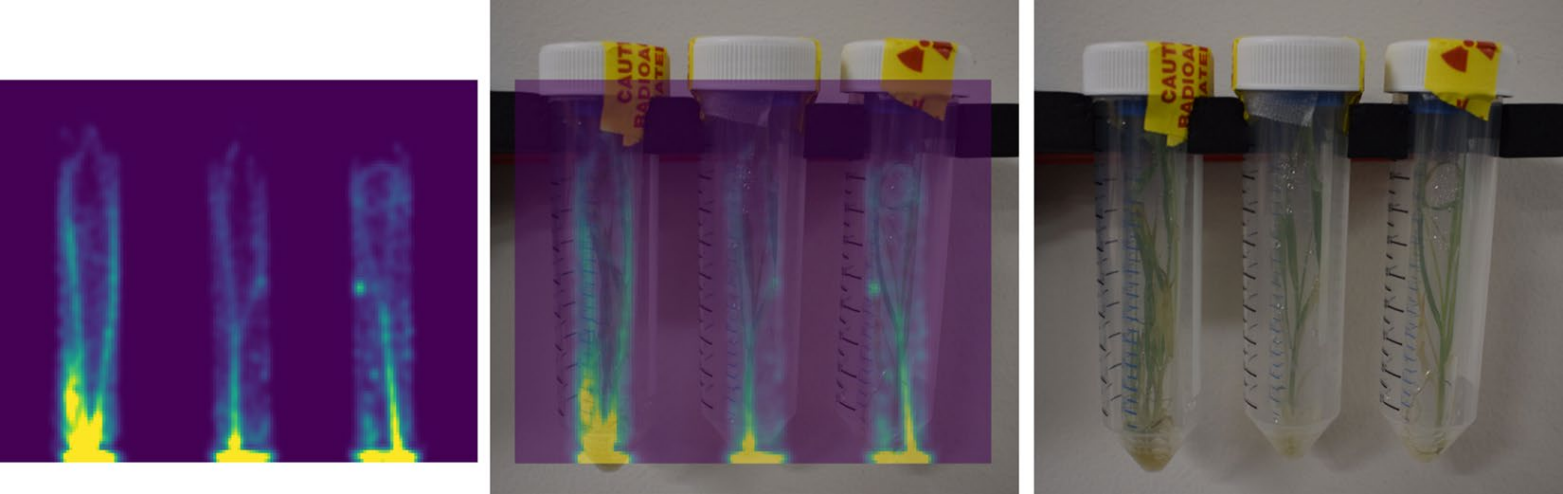
Co-registration of reconstructed PET images and physical plant structures in the shoot (sheath and leaf blades). This example shows a transverse slice through the center of three A10.1 plants imaged at *t* = 10 days (left), the same images overlaid with a registered photograph of the plants taken at the same time point (center), and the raw photograph (right).

Figure 4 shows a representative example of the co-registered PET and photographic images, confirming that the bio-distribution of ^22^Na closely matched the physical structures of the plant shoot (sheath and leaf blades). As expected, we observed no PET activity in dead leaves. The inner tube wall is apparent in the reconstructed PET images (Figs. 3 and 4), being clearest at later time points when sodium had travelled further into the shoot. This results from a small percentage of emitted positrons escaping the thin plant leaves and depositing energy in the wall of the tube. The resulting spill-over effect spreads the true activity at a given height of the plant over a region proportional to the separation of the tube wall and plant stem. Spill-over was partially compensated for by including the tube wall in the ROI analysis.

### Uptake dynamics quantification

**Figure 5:**
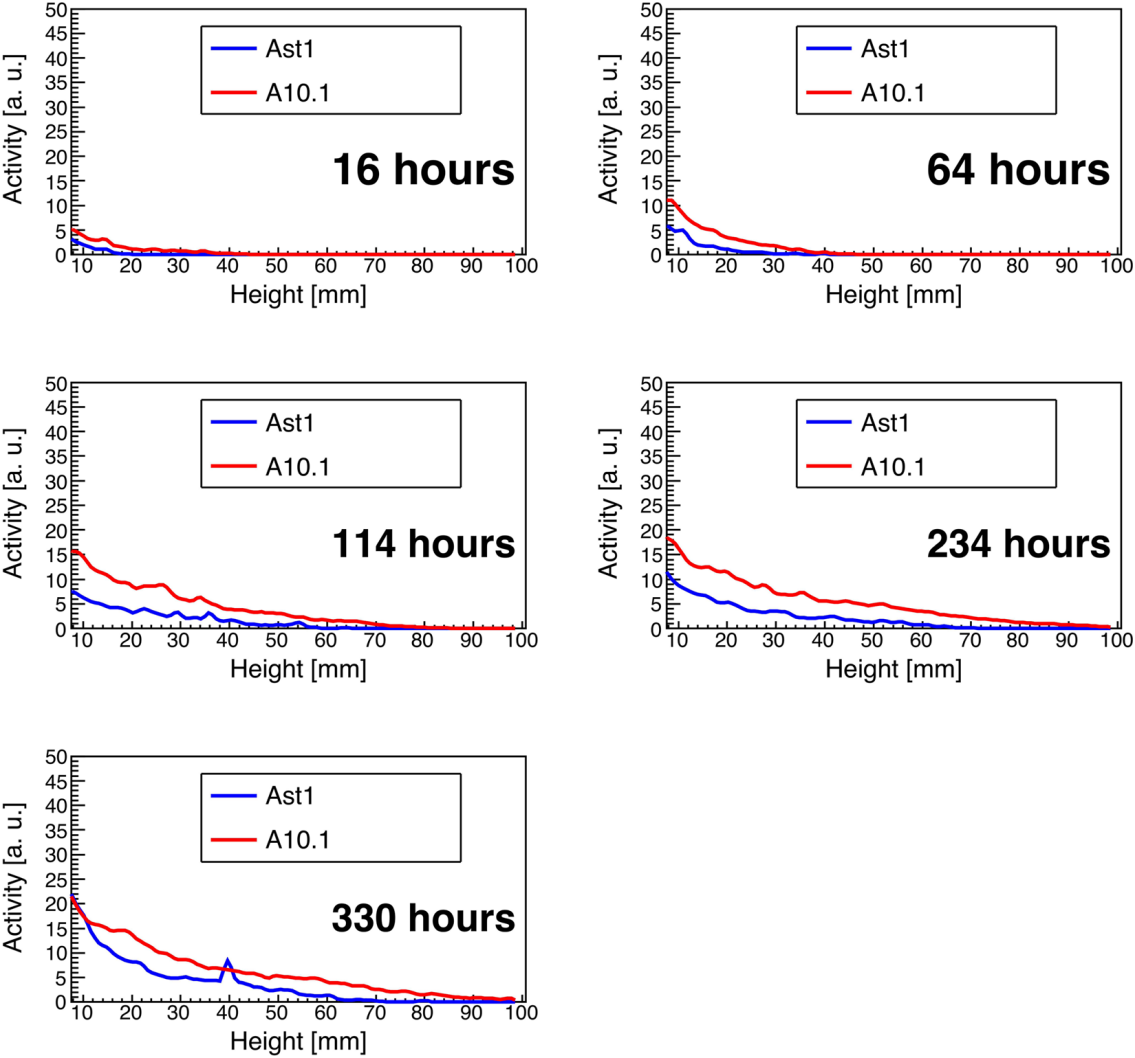
^22^Na activity as a function of height along the stem. The red and blue curves correspond to the A10.1 and Ast1 accessions, respectively.

**Figure 6:**
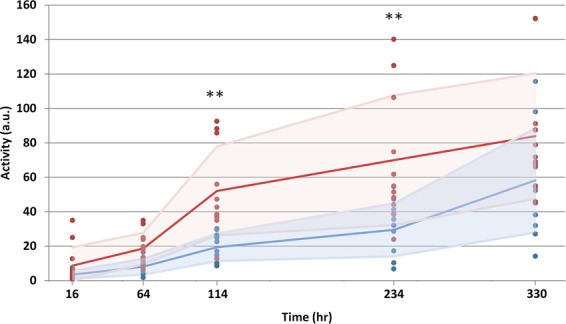
Dynamic sodium transport. The red and blue markers represent the A10.1 and Ast-1 accessions, respectively. Solid lines show the average trend for each accession. The shaded regions represent +/− 1 standard deviation. ** indicates high statistical significance (p < 0.01).

Figures 5 and 6 capture the dynamic behavior of salt transport in the two accessions of green millet based on the quantitative ROI analysis. Note that for both figures, data from the first 10 slices (corresponding to the roots) was excluded to better compare the relative uptake in the shoot, and to avoid possible bias from image reconstruction artifacts that can manifest at the edge of the FoV. Figure 5 shows the average ^22^Na uptake for each accession as a function of height along the stem and indicates that the A10.1 accession transported roughly twice as much sodium as the Ast-1 accession. Sodium was also transported at a faster rate in the A10.1 accession, moving through the entire plant within 1 week. By comparison, sodium had not reached the full height of the Ast-1 plants after 2 weeks.

Figure 6 shows the total ^22^Na uptake for each individual plant specimen at each time point. There was a highly significant difference in the total uptake at *t* = 114 h (p = 4 × 10^−4^) and *t* = 234 h (p = 3 × 10^−3^), confirming that the rate of uptake of the A10.1 accession was greater than that of the Ast-1 accession. This was further supported by the ANOVA results which showed a highly significant correlation (p = 0.012) for the interaction (time × accession).

### Sodium content and gene expression analysis

**Figure 7:**
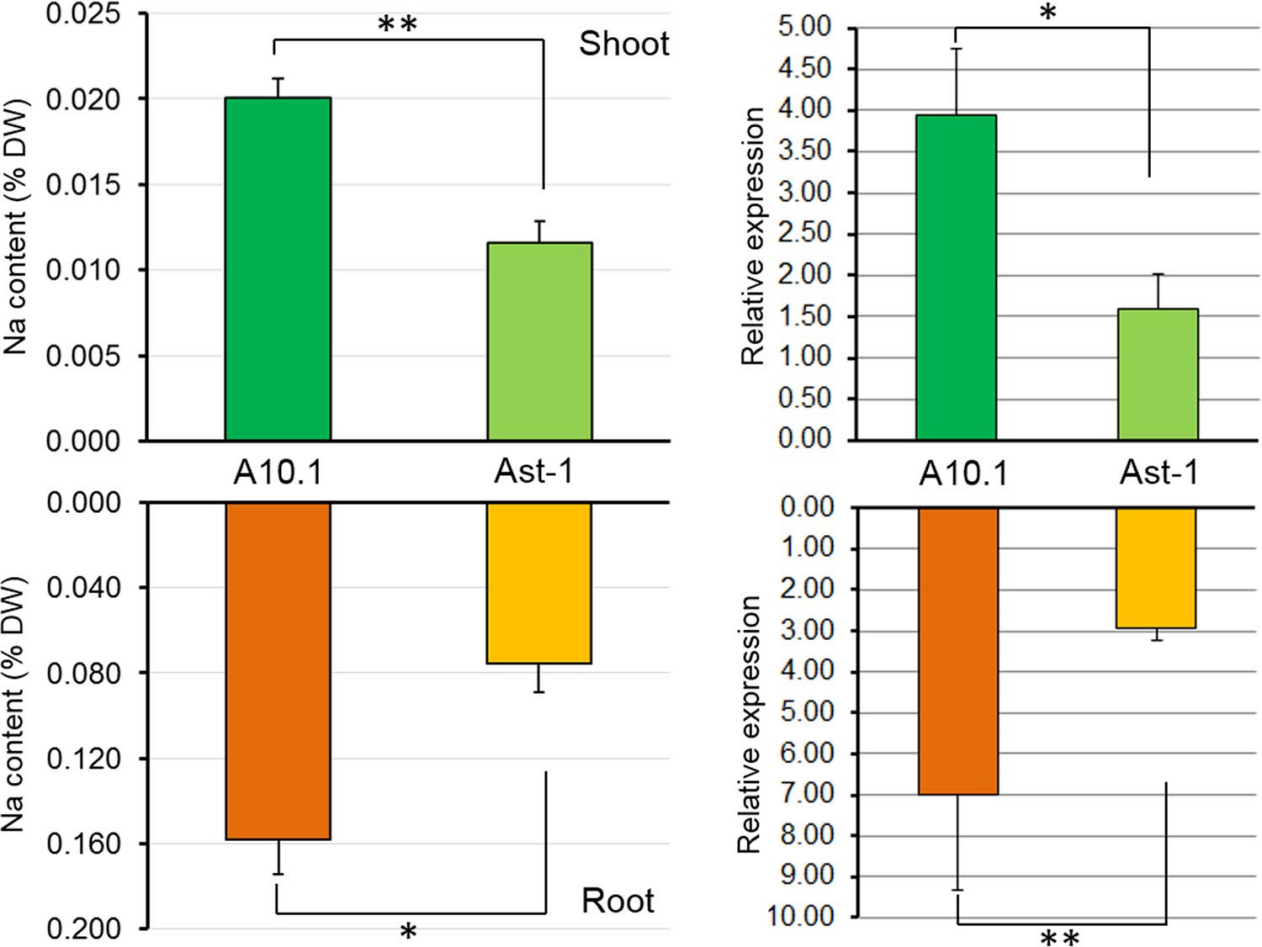
Na^+^ content (left) and NHX gene expression (right) of shoot (top) and roots (bottom) in *S. viridis* accessions A10.1 and Ast-1. The bar graphs on the left represent mean percentage (%) dry weight (DW) ± standard error (SE) of Na^+^ content after 10 d (n = 50). The bar graphs on the right represent mean relative expression ± SE of *NHX* transcripts after 10 d (n = 50). The statistical analysis used  the Student’s *t*-test; significant differences are indicated for p < 0.05 (*) and p < 0.01 (**).

Figure 7-left and 7-right show the Na^+^ content as a percentage of dry weight and the relative expression of the *NHX* gene, respectively. In both cases, Na^+^ content and *NHX* expression were greater for the A10.1 accession than the Ast-1 accession. These differences were statistically significant and consistent across both roots and shoots.

## Discussion

In this study, we investigated the feasibility of using PET imaging to study dynamic salt transport in intact plants, with a particular focus on the type of information that can be obtained, how this augments current techniques, and practical imaging aspects.

### Utility of PET for sodium transport research in the plant sciences

Our results clearly demonstrate the utility of PET for observing and quantifying dynamic sodium transport in intact plants over a 2-week period. This supports the findings of Fujimaki *et al.* who studied common reed using PET^26^. A notable feature of the dynamic behavior we observed is the long timescale of sodium transport; it took >1 week for sodium to fully distribute throughout the shoots of the seedlings. Detection sensitivity is another key feature of PET and one which is vital for this application. Our results demonstrate detection of sub-nanomolar concentrations of Na^+^ in the shoot (the concentration of hot Na^+^ in the incubation solution was ~3.6 nM). Compared to ^23^Na MRI^35,36^), PET sensitivity is several orders of magnitude better.

There was excellent agreement between the quantitative PET measurements and the Na^+^ content snapshot (day 10). Unfortunately, it was not possible to perform an absolute comparison of the PET data with the Na^+^ content measurements since we could not obtain the dry weight of the seedlings used in the PET experiments at intermediate time points. Therefore, further tests are required to fully validate the absolute quantification of PET in this application.

In previous work it was shown that the Ast-1 accession is extremely sensitive to water and heat stress and that the A10.1 accession is relatively insensitive^27^. Our PET results corroborate the fact that there are statistically significant genetic-based differences between these accessions. Unlike^27^, the present study involved non-stress conditions. It is notable, however, that significant differences exist even in the baseline (non-stress) condition. We would expect the same trends to be mirrored in the stress condition – although this remains to be tested in a future study. The increased expression of the *NHX* gene we measured in the A10.1 accession provides a plausible explanation for the increased sodium transport we observed for this genotype based on both PET and Na^+^ content measurements.

Previous studies of salt transport in related crop species indicate that increased salt transport is associated with relative sensitivity to salt stress (i.e. salt intolerance). Conversely, reduced transport, for example through active exclusion of sodium from the root, is associated with relative salt tolerance (e.g.^13^). Therefore, notwithstanding the fact that we tested the plants in a stress-free scenario, our results would suggest that the Ast-1 accession is more salt tolerant and the A10.1 accession is more salt-sensitive. This conclusion is rather unexpected given the reverse findings for these accessions for water and heat stress^27^. It suggests that sensitivity to one type of stress does not imply sensitivity to another. Further, we hypothesize that the Ast-1 accession has mechanisms (such as reduced expression of sodium transport proteins arising from transcripts like *NHX*) to better regulate salt intake via the roots and throughout the plant compared to the A10.1 accession, which also explains its reduced dynamic transport of salt over the 2-week period we observed. Further investigation is required to properly test this hypothesis and to understand how the salt transport behavior relates to the underlying mechanisms of salt regulation at entry (roots) and distribution throughout the plant stem. Our present study provides the proof-of-principle that such future studies involving PET are well justified.

### ^22^Na PET imaging hardware

Nearly all previously published plant PET studies have involved custom-designed scanners developed in-house. Here we repurposed the Inveon PET scanner, which is used routinely in our imaging center for small animal studies. The high spatial resolution (~1.5 mm) of the Inveon suits imaging of small seedlings and the FoV comfortably supports imaging of at least three seedlings simultaneously. The PET system in^24^ was also based on the Inveon, however the authors reconfigured the detector modules into an entirely new geometry better suited to imaging large plants. As a result, the spatial resolution and sensitivity were both poorer compared to the native scanner used in our study. Although the Inveon is well-suited to imaging seedlings, it is not appropriate for large plants. In this case it may be better to use a clinical PET scanner with bore diameter close to 60 cm or, alternatively, a custom PET system where the detectors translate on a linear stage (e.g.^24^).

The most closely related work to what we present here is that of Fujimaki *et al*.^26^. There, PET was used successfully to corroborate the known blockade to Na transport in common reed, and to compare this with rice in which no such blockade has been demonstrated. Imaging was performed using PETIS, an opposing two-panel PET system with a large FoV suitable for larger plants. Since PETIS lacks a complete detector ring (i.e. is limited-angle), it is only suitable for reconstructing objects in the mid focal plane using a simplified 2D image reconstruction; out-of-plane spatial resolution for this system is very poor. Despite the limitations of PETIS, this study provides further recent support that PET can complement more traditional quantitative molecular techniques in the plant sciences.

### PET quantification

One factor affecting quantification in plant PET studies, especially those involving seedlings and thin plant tissues such as leaves, is positrons exiting the tissues before annihilation. In our study this resulted in weak signal from the wall of the inner tube containing the plant (Figs. 3 and 4). Other authors have reported this issue and proposed various methods to address it. One example is the use of plastic cuvettes between which leaves are sandwiched and which also serve to contain radiolabeled CO_2_ delivered to the leaves^20^. In^37^, the PhytoPET imaging system was developed to detect emitted beta particles (positrons and electrons) directly rather than gamma photons from an annihilation event. However, this system is not tomographic and the beta probe can only provide count rates at specific locations along the plant. In our case, the spread of measured activity into the tube wall represents a convolution of the true distribution; thus, deconvolution approaches, akin to the correction of partial volume effects which have been explored in detail in PET-based imaging, are certainly applicable to improve quantification.

### Practicality and radiation safety

The literature on PET imaging of plants using ^22^Na is extremely scant. One reason for this may be the challenges associated with handling unsealed long-lived radioisotopes in the laboratory to ensure a low risk of contamination. Here we have described a robust ^22^Na PET imaging protocol to study sodium transport in plants. We have paid particular attention to developing a practical protocol for plant incubation and imaging which can be performed with low risk. The rinsing step in our procedure (just prior to imaging) carries the greatest risk of contamination; however, this step is greatly simplified by retaining the seedlings permanently inside the smaller tube.

### Study limitations

In our study, the total concentration of sodium in the incubation medium was too low to cause salt stress. Thus, our data pertain to transport behavior under favorable conditions. It is possible that the relative response of the accessions may differ under stress conditions, though this seems unlikely. At any rate, an obvious follow-up study is to perform PET imaging on the plants when subjected to salt stress.

There was considerable variability among plants of the same variety at the same time point in our experimental data. Although this did not prevent statistical differences in sodium transport from being identified in our study, variability could be reduced, along with the number of plants, by tighter control of environmental conditions such as lighting and temperature, and by avoiding multiple rinsing steps. Multiple rinsing steps can be avoided by using an alternative incubation regime in which plants are first subjected to a ‘hot’ growth medium for a set time before being transferred to a ‘cold’ growth medium for the remainder of the experiment^25^. Moreover, making the cold growth medium free of salt would allow us to observe reversible transport of sodium back down the stem towards the roots, something that was not possible to observe in the current study since sodium was constantly available in the incubation medium over the 2-week period.

The Inveon scanner is limited to imaging plants with a maximum height of ~120 mm. To accommodate larger or soil-bound plants, custom-built PET systems with the detector ring axis aligned vertically (e.g.^20,24^) have distinct advantages, albeit with potentially poorer spatial resolution and sensitivity specifications. Our future work will consider the feasibility of a long-axis, high resolution PET system for large plants.

## Summary and Conclusions

We have imaged the three-dimensional dynamic transport of sodium in different genotypes of *S. viridis* for the first time using a repurposed small animal PET scanner. Our preliminary data suggest that PET imaging can be used to study salt transport dynamics and regulation in intact plants over physiologically relevant timescales and with high sensitivity. This is not possible with currently available techniques. The main contributions of this study are:

(i) We have described a safe ^22^Na PET imaging protocol to study sodium transport in plants using existing high-resolution PET technology that is readily available in many imaging centers.
(ii) We have demonstrated the use of PET to provide quantitative data relating to the dynamic uptake and transport of Na^+^ in seedlings, including detection of significant differences in salt transport for different accessions.
(iii) We have independently validated the observed differences from PET using conventional chemical and genetic techniques in widespread use in the plant sciences.
(iv) And we have shown evidence that PET may provide an efficient screening method for relative salt tolerance in different plant varieties, using green millet as a test case.

In conclusion, this study adds further support to the findings in^26^ that PET is a useful complement to existing techniques for understanding sodium transport and regulatory mechanisms in plants, and that it may provide a useful screening tool for research in this area. This study paves the way for more targeted experiments that utilize PET to tease out the dynamics of these regulatory mechanisms at a systems level.
